# Supplementary material for: An Efficient Feature Selection Algorithm for Gene Families Using NMF and ReliefF
Source: Genes (Basel). 2023 Feb 6;14(2):421. doi: 10.3390/genes14020421 (PMC9957060; doi:10.3390/genes14020421)
Supplement: Supplementary file 1 [file genes-14-00421-s001.zip › Supplementary File S1.pdf]

Supplementary File S1. Performance comparison under different feature number.

Number of features is 5, SVM

	ReliefF	SVM-RFE	PCA-ReliefF	NMF-ReliefF	Baseline
ACC	0.5533	0.7633	0.7818	0.6707	0.6833
SEN	0	0.4833	0.5833	0.6595	0.5667
SPE	1	0.9500	0.9381	0.9131	0.7833
AUC	0.4375	0.6583	0.7059	0.8592	0.6292

Number of features is 5, RF

	ReliefF	SVM-RFE	PCA-ReliefF	NMF-ReliefF	Baseline
ACC	0.5478	0.7400	0.6818	0.6455	0.6633
SEN	0.6917	0.5667	0.5833	0.5310	0.6000
SPE	0.8667	0.8667	0.7524	0.7976	0.8000
AUC	0.6958	0.6833	0.7059	0.6357	0.5875

Number of features is 5, KNN

	ReliefF	SVM-RFE	PCA-ReliefF	NMF-ReliefF	Baseline
ACC	0.7833	0.6800	0.6654	0.7436	0.6433
SEN	0.6917	0.5833	0.5167	0.3810	0.7500
SPE	0.8500	0.7417	0.7857	0.5083	0.7250
AUC	0.6958	0.6333	0.7059	0.7119	0.6292

Number of features is 10, SVM

	ReliefF	SVM-RFE	PCA-ReliefF	NMF-ReliefF	Baseline
ACC	0.4273	0.7867	0.8036	0.8912	0.6833
SEN	0.2000	0.6500	0.6333	0.7876	0.5667
SPE	0.8000	0.9500	0.9428	0.8700	0.7833
AUC	0.4333	0.7167	0.7381	0.8198	0.6292

Number of features is 10, RF

	ReliefF	SVM-RFE	PCA-ReliefF	NMF-ReliefF	Baseline
ACC	0.5548	0.7467	0.6654	0.6691	0.6633
SEN	0.7433	0.6500	0.5833	0.5705	0.6000
SPE	0.9464	0.9000	0.7405	0.8200	0.8000
AUC	0.8331	0.6750	0.5774	0.6928	0.5875

Number of features is 10, KNN

	ReliefF	SVM-RFE	PCA-ReliefF	NMF-ReliefF	Baseline
ACC	0.8236	0.6433	0.7018	0.7418	0.6433
SEN	0.9064	0.5500	0.5333	0.4705	0.7500
SPE	0.7417	0.7583	0.8595	0.3967	0.7250
AUC	0.7915	0.5417	0.6214	0.7578	0.6292

Number of features is 20, SVM

	ReliefF	SVM-RFE	PCA-ReliefF	NMF-ReliefF	Baseline
ACC	0.4273	0.7867	0.8018	0.8433	0.6833
SEN	0.2000	0.6250	0.6543	0.6300	0.5667
SPE	0.8000	0.9333	0.9666	0.9267	0.7833
AUC	0.4670	0.6708	0.7263	0.7750	0.6292

Number of features is 20, RF

	ReliefF	SVM-RFE	PCA-ReliefF	NMF-ReliefF	Baseline
ACC	0.7442	0.7100	0.5818	0.6473	0.6633
SEN	0.7933	0.6000	0.6443	0.5267	0.6000
SPE	0.8529	0.8583	0.5817	0.7095	0.8000
AUC	0.7552	0.6458	0.5414	0.6177	0.5875

Number of features is 20, KNN

	ReliefF	SVM-RFE	PCA-ReliefF	NMF-ReliefF	Baseline
ACC	0.8055	0.6300	0.6836	0.7455	0.6433
SEN	0.7433	0.4833	0.6757	0.4633	0.7500
SPE	0.8500	0.7500	0.7550	0.3571	0.7250
AUC	0.7290	0.5292	0.6330	0.7573	0.6292

Number of features is 30, SVM

	ReliefF	SVM-RFE	PCA-ReliefF	NMF-ReliefF	Baseline
ACC	0.7667	0.7800	0.7054	0.8410	0.6833
SEN	0.4667	0.5750	0.5300	0.7162	0.5667
SPE	1	0.9333	0.8533	0.9250	0.7833
AUC	0.6667	0.6917	0.6160	0.8510	0.6292

Number of features is 30, RF

	ReliefF	SVM-RFE	PCA-ReliefF	NMF-ReliefF	Baseline
ACC	0.7600	0.6233	0.6836	0.6855	0.6633
SEN	0.7250	0.4417	0.6500	0.7962	0.6000
SPE	0.7667	0.7500	0.7200	0.6750	0.8000
AUC	0.6917	0.5472	0.6353	0.7398	0.5875

Number of features is 30, KNN

	ReliefF	SVM-RFE	PCA-ReliefF	NMF-ReliefF	Baseline
ACC	0.8000	0.6833	0.6436	0.7018	0.6433
SEN	0.6667	0.5667	0.5300	0.4638	0.7500
SPE	0.9083	0.8000	0.7467	0.3233	0.7250
AUC	0.7417	0.5889	0.5967	0.7152	0.6292

Number of features is 40, SVM

	ReliefF	SVM-RFE	PCA-ReliefF	NMF-ReliefF	Baseline
ACC	0.7867	0.7867	0.6673	0.8270	0.6833
SEN	0.5417	0.6833	0.5600	0.8433	0.5667
SPE	0.9667	0.8917	0.7448	0.8743	0.7833
AUC	0.6917	0.7333	0.6360	0.8832	0.6292

Number of features is 40, RF

	ReliefF	SVM-RFE	PCA-ReliefF	NMF-ReliefF	Baseline
ACC	0.7833	0.7300	0.6091	0.7236	0.6633
SEN	0.6750	0.6750	0.5267	0.6967	0.6000
SPE	0.9167	0.8250	0.6943	0.7657	0.8000
AUC	0.7500	0.7083	0.5939	0.8122	0.5875

Number of features is 40, KNN

	ReliefF	SVM-RFE	PCA-ReliefF	NMF-ReliefF	Baseline
ACC	0.7667	0.7700	0.6691	0.7455	0.6433
SEN	0.7583	0.7083	0.5733	0.4433	0.7500
SPE	0.8333	0.8667	0.7495	0.3705	0.7250
AUC	0.7000	0.7250	0.6539	0.7888	0.6292

Number of features is 50, SVM

	ReliefF	SVM-RFE	PCA-ReliefF	NMF-ReliefF	Baseline
ACC	0.7682	0.7467	0.7091	0.8128	0.6833
SEN	0.5567	0.5500	0.5962	0.7400	0.5667
SPE	0.9333	0.8917	0.8000	0.9114	0.7833
AUC	0.6317	0.6250	0.6589	0.8241	0.6292

Number of features is 50, RF

	ReliefF	SVM-RFE	PCA-ReliefF	NMF-ReliefF	Baseline
ACC	0.7267	0.7100	0.7636	0.7309	0.6633
SEN	0.5767	0.4667	0.6562	0.7333	0.6000
SPE	0.8333	0.8667	0.8450	0.7762	0.8000
AUC	0.5517	0.5833	0.7222	0.7655	0.5875

Number of features is 50, KNN

	ReliefF	SVM-RFE	PCA-ReliefF	NMF-ReliefF	Baseline
ACC	0.7589	0.6900	0.6855	0.7473	0.6433
SEN	0.7767	0.5333	0.6629	0.4367	0.7250
SPE	0.8500	0.7500	0.6933	0.4038	0.7500
AUC	0.7267	0.5667	0.6685	0.7289	0.6292
